# Supplementary material for: Discordant Genome Assemblies Drastically Alter the Interpretation of Single-Cell RNA Sequencing Data Which Can Be Mitigated by a Novel Integration Method
Source: Cells. 2022 Feb 10;11(4):608. doi: 10.3390/cells11040608 (PMC8870202; doi:10.3390/cells11040608)
Supplement: Supplementary file 1 [file cells-11-00608-s001.zip › cells-1559420-supplementary.pdf]

3′ UTR Extension

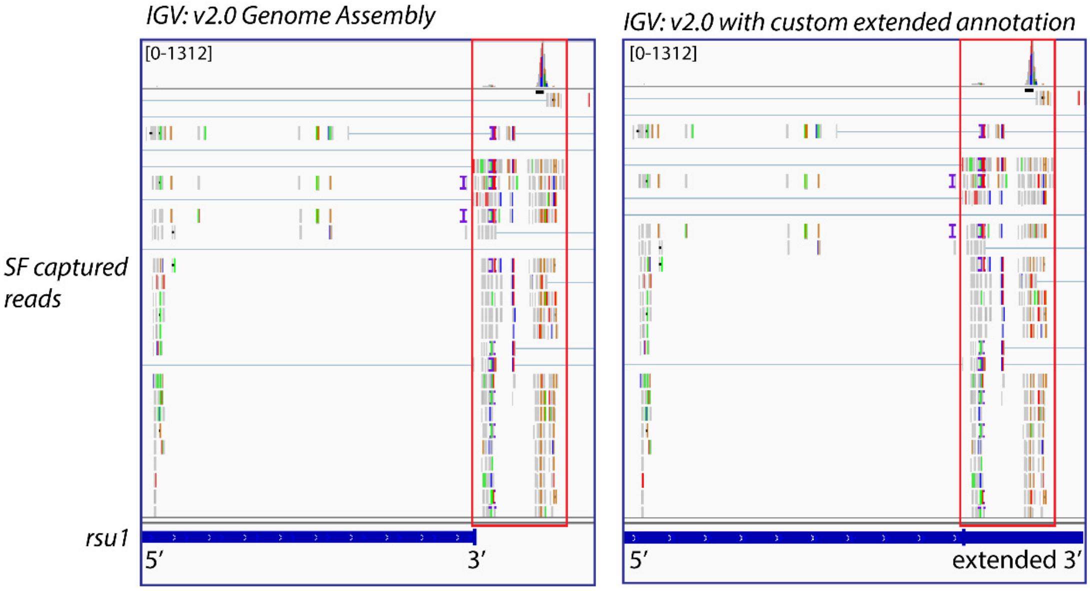

Supplementary Figure S1: Custom 3′ UTR extension enables transcript capture for genes with incomplete 3′UTR annotation. IGV browser was used to compare aligned reads from the possorted_genome_bam.bam.file using v2.0 gtf file available from Ensembl (v103) and our the custom extended gtf file.

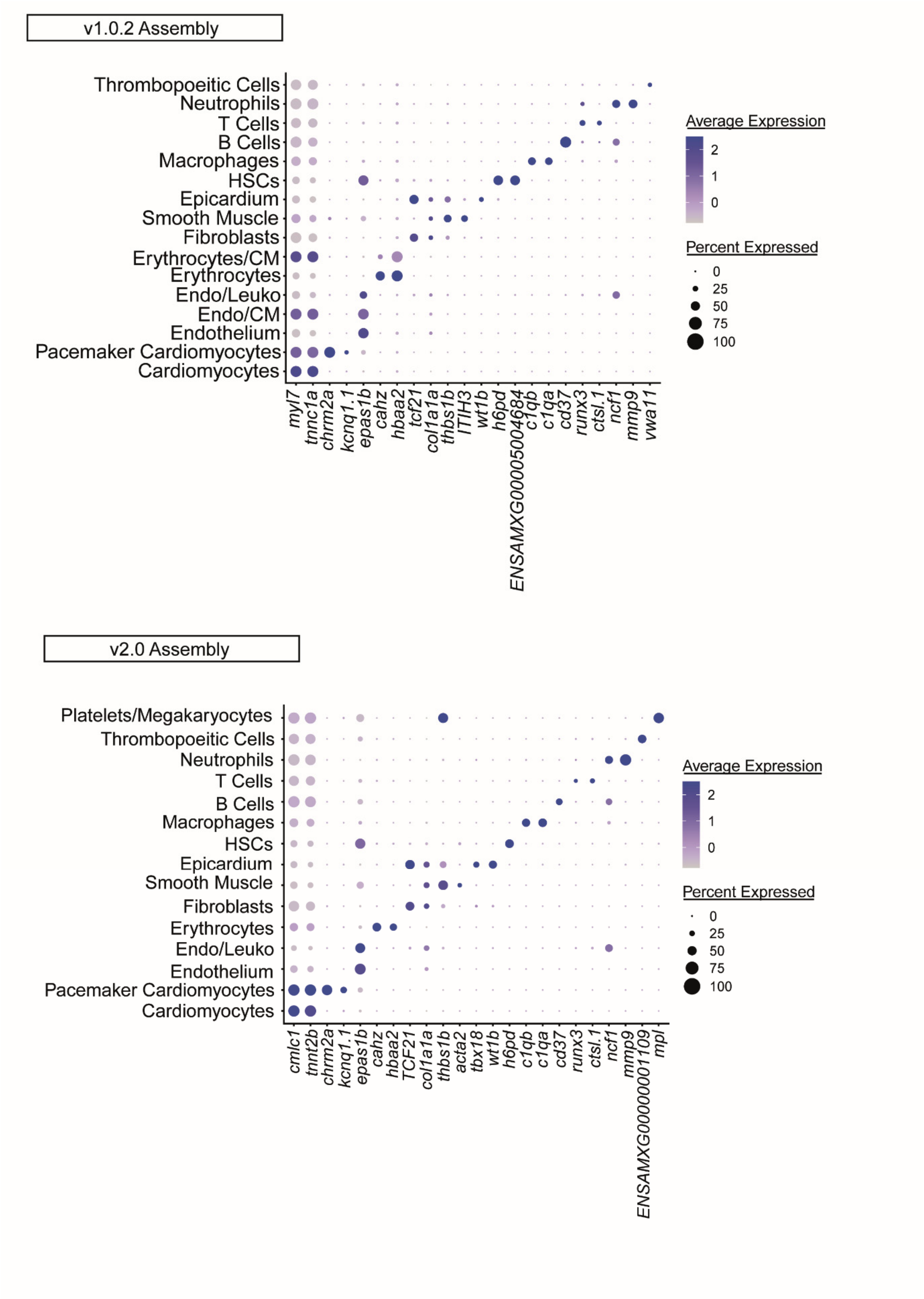

Supplementary Figure S2: Identification of the *A. mexicanus* major cardiac cell types. DotPlot showing example genes used to determine the identity of the different cell clusters when aligned to the respective v1.0.2 and v2.0 assemblies.

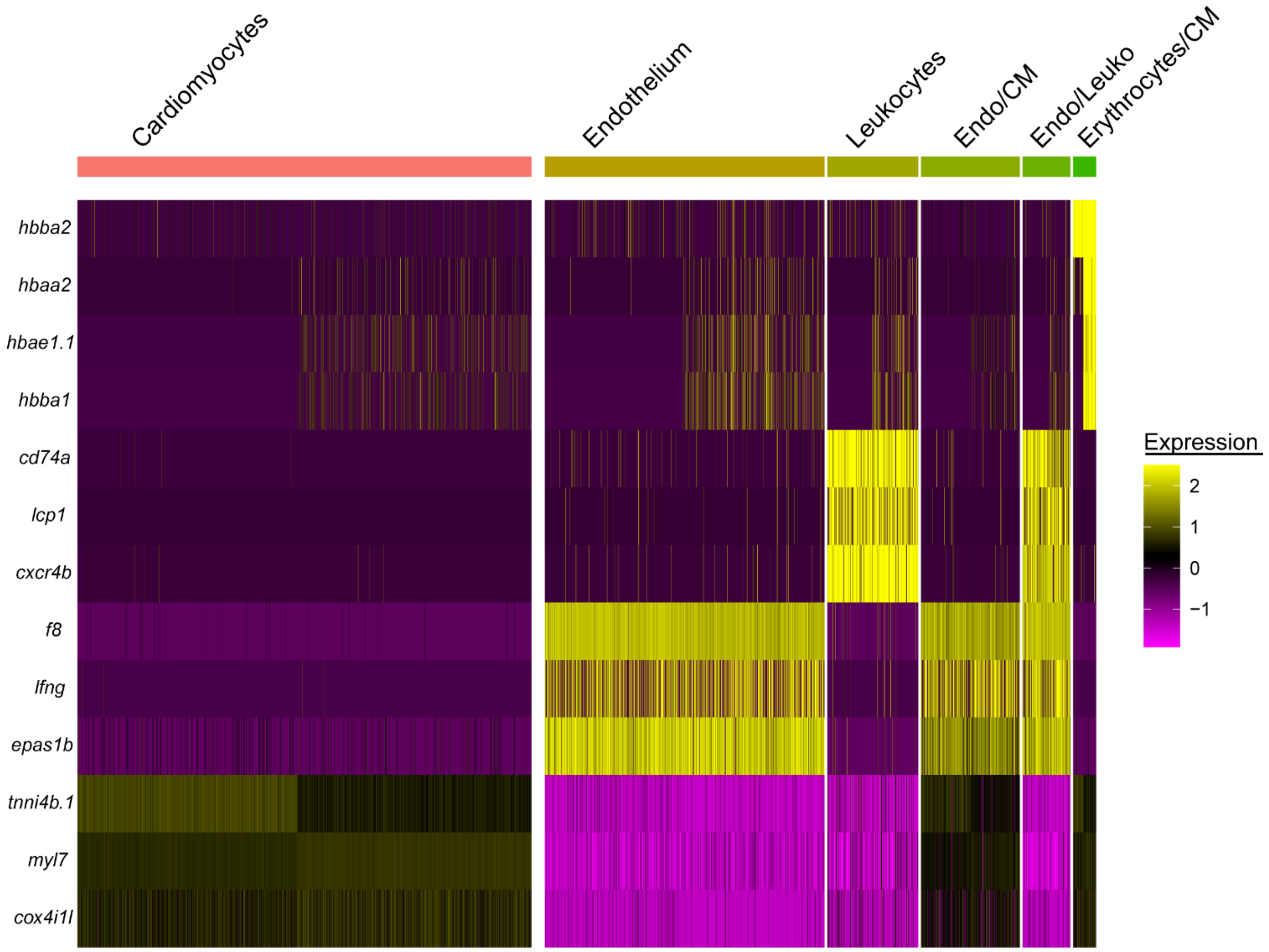

Supplementary Figure S3: The integrated dataset enabled more accurate identification of doublets. Heatmap of the transcriptional profile of identified doublet clusters showing that cells from each doublet cluster expressed top genes from multiple cardiac cell types. Cardiomyocytes: cluster 0; Endothelium: cluster 5; Leukocytes: cluster 20; Endo/CM: cluster 18; Endo/Leuko: cluster 23; Erythrocytes/CM: cluster 27

Supplementary Table S1: Consistent Gene Symbol Markers for the major cardiac cell types. The listed genes are the results of FindConservedMarkers, representing cell type markers that are present with the same gene symbol in both genome assemblies. We present this list of genes as a tool for other *A. mexicanus* researchers that can be used, in addition to the genome assembly-specific cell markers to enable accurate identification of cardiac cell types.

Cell Type	Consistent Markers
**Cardiomyocytes**	*mia*<br>*ndufc2*<br>*ckma*<br>*cox4i1l*<br>*ndufb4*<br>*sat1b*<br>*csrnp1b*<br>*hadhb*<br>*zgc:195173*<br>*NDUFB1*<br>*COX7A2*<br>*msna*<br>*jdp2b*<br>*fosab*<br>*lyrm2*<br>*zgc:86709*<br>*myct1a*<br>*akap12b*<br>*pdgfra*<br>*kdr*
**B cells**	*cd37*<br>*zgc:194275*<br>*LYN*<br>*si:dkey-24p1.1*<br>*cxcr4b*<br>*ncf1*<br>*cd74a*<br>*lyn.1*<br>*ikzf1*<br>*p2ry10*<br>*si:dkey-88e18.2*<br>*rel*<br>*rgs13*<br>*gpr18*<br>*laptm5*<br>*pfn1*<br>*cst3*<br>*pik3cd*

	dusp2<br>*mb*
**Endothelium**	*ndufc2*<br>*ckma*<br>*cox4i1l*<br>*ndufb4*<br>*wasf3b*<br>*csrnp1b*<br>*hadhb*<br>*SBSPON*<br>*zgc:195173*<br>*NDUFB1*<br>*msna*<br>*fosab*<br>*hpcal1*<br>*zgc:86709*<br>*myct1a*<br>*akap12b*<br>*pdgfra*<br>*kdr*<br>*hspa1b*<br>*ppdpfa*

**Epicardium**	*wt1b*<br>*sftpba*<br>*nol4lb*<br>*stmn1a*<br>*lcp1*<br>*lsp1b*<br>*rgs13*<br>*flt3*<br>*unc93b1*<br>*cd74a*<br>*rnaset2l*<br>*cxcr4b*<br>*laptm5*<br>*zgc:66024*<br>*arpc1b*<br>*npr3*<br>*PTPRC*<br>*spi1b*<br>*zgc:194312*<br>*colec11*
**Fibroblasts**	*pltp*<br>*thbs1a*<br>*col12a1a*<br>*ccl25b*<br>*c6*<br>*flnb*<br>*rbp4*<br>*hmx4*<br>*clec19a*<br>*fbln1*<br>*ebf3a.1*<br>*dcn*<br>*si:ch1073-459j12.1*<br>*apoeb*<br>*nkx3.3*<br>*mdka*<br>*krt18b*<br>*clu*<br>*CPZ*<br>*pamr1*

**Haematopoietic Stem Cells**	*efnb1*<br>*slc16a10*<br>*igfbp1a*<br>*h6pd*<br>*rgs5a*<br>*plvapa*<br>*cpb1*<br>*igfbp7*<br>*CASQ2*<br>*meox2a*<br>*meox1*<br>*tbx3a*<br>*slco1d1*<br>*slc26a10*<br>*si:ch211-251b21.1*<br>*gpr156*<br>*ext1c*<br>*PRSS23*<br>*abcb4*<br>*ca16b*
**Pacemaker Cardiomyocytes**	*ckbb*<br>*sema3bl*<br>*chrm2a*<br>*pfklb*<br>*KCNH7*<br>*ptn*<br>*smtnl1*<br>*kcnq1.1*<br>*myoz1a*<br>*col18a1a*<br>*fbn2a*<br>*sema3fb*<br>*tnnt3b*<br>*st3gal2*<br>*tnnc1b*<br>*tbx5a*<br>*nxt2*<br>*twf2b*<br>*lgals2a*<br>*EPHB3*

**Macrophages**	*si:dkey-5n18.1*<br>*MPEG1*<br>*serping1*<br>*f13a1b*<br>*PTPRC*<br>*c1qb*<br>*c1qc*<br>*c1qa*<br>*ETS2*<br>*pfn1*<br>*RAB20*<br>*spi1a*<br>*itgam*<br>*cxcl8a*<br>*card9*<br>*cxcr4b*<br>*csf1ra*<br>*cd74a*<br>*lcp1*<br>*spi1b*
**Neutrophils**	*gramd1c*<br>*adam8a*<br>*alox5ap*<br>*cebp1*<br>*ltb4r*<br>*c6ast1*<br>*st8sia4*<br>*slc2a3b*<br>*mmp25b*<br>*s1pr4*<br>*gpr84*<br>*cass4*<br>*acsl4b*<br>*card9*<br>*prom2*<br>*wu:fj16a03*<br>*ccdc88b*<br>*fut7*<br>*cyba*<br>*tprg1*

**Platelets/Megakaryocytes**	*mpl*<br>*ly97.3*<br>*si:ch73-309g22.1*<br>*zgc:86896*<br>*itprid1*<br>*si:ch211-103n10.5*<br>*btk*<br>*itga2.2*<br>*gata1a*<br>*fermt3b*<br>*si:ch211-234h8.7*<br>*plac8l1*<br>*dennd2c*<br>*thbs1b*<br>*scinla*<br>*pfn1*<br>*blk*<br>*si:busm1-57f23.1*<br>*pfkfb3*<br>*f2r*
**Erythrocytes**	*si:ch211-103n10.5*<br>*wu:fj16a03*<br>*slc7a8b*<br>*mgst1.2*<br>*plac8l1*<br>*nt5c2l1*<br>*gp9*<br>*cahz*<br>*nmt1b*<br>*slc4a1a*<br>*epor*<br>*rhag*<br>*gata1a*<br>*rex1bd*<br>*blvrb*<br>*tmod4*<br>*fam117ab*<br>*creg1*<br>*ybx1*<br>*slc25a5*

**Thrombopoeitic Cells**	*ca4b*<br>*cxcr2*<br>*ankrd22*<br>*f5*<br>*ly97.3*<br>*cass4*<br>*zgc:136605*<br>*wasb*<br>*sla1a*<br>*cnr2*<br>*gpr183a*<br>*stx11a*<br>*CKLF*<br>*fut7*<br>*ltb4r*<br>*plek*<br>*arhgap15*<br>*wu:fj16a03*<br>*scin*<br>*lsp1b*
